# Supplementary material for: Influence of the Nature of Aminoalcohol on ZnO Films Formed by Sol-Gel Methods
Source: Nanomaterials (Basel). 2023 Mar 15;13(6):1057. doi: 10.3390/nano13061057 (PMC10052816; doi:10.3390/nano13061057)
Supplement: Supplementary file 1 [file nanomaterials-13-01057-s001.zip › nanomaterials-2230109-supplementary.pdf]

Supplementary Materials

Influence of the nature of the aminoalcohol on ZnO films formed by sol-gel methods

Figures S1–S4: Indexed XRD patterns, in the main angular range from 25 to 75 deg 2θ, of the ZnO films **1a–1d** obtained at 400, 500, and 600 °C.**;**

Figure S5: Indexed XRD patterns, in the main angular range from 25 to 75 deg 2θ, of the ZnO films **1e** obtained at 300, 400, 500, and 600 °C.**;**

Figure S6: Variation of the relative abundance of the sphalerite and wurtzite forms of ZnO present in films **1a–1e** obtained after the thermal treatment at T = 400, 500 and 600 °C**;**

Figures S7–S10: Tauc's plots for films **1a- 1c** and **1e** at each annealing temperature;

Figures S11–S14: Fittings to find the Urbach's energy of films **1a–1c** and **1e** at each annealing temperature;

Figures S15–S18: PL spectra of films **1a–1c** and **1e** annealed at 600 °C and Gaussian deconvolution of their DLE bands.

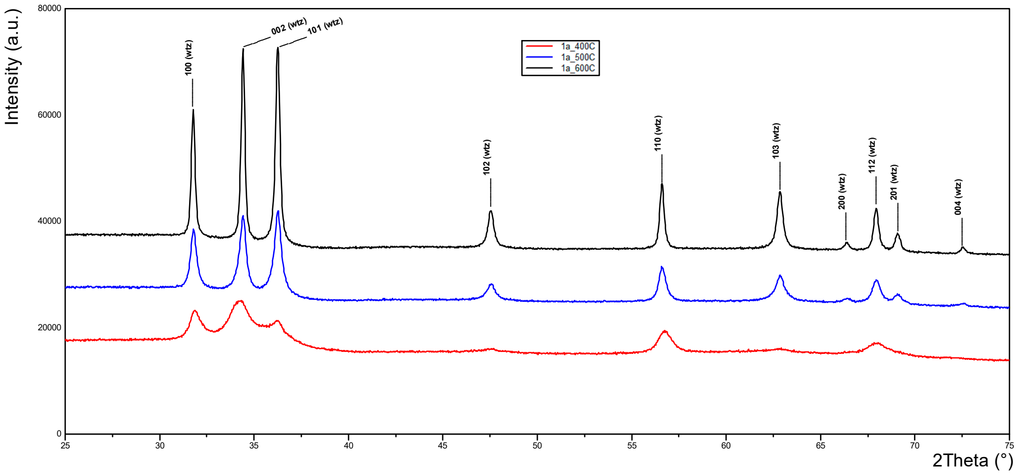

Figure S1: Indexed XRD patterns, in the main angular range from 25 to 75 deg 2θ, of the ZnO films **1a** obtained at 400, 500, and 600 °C.

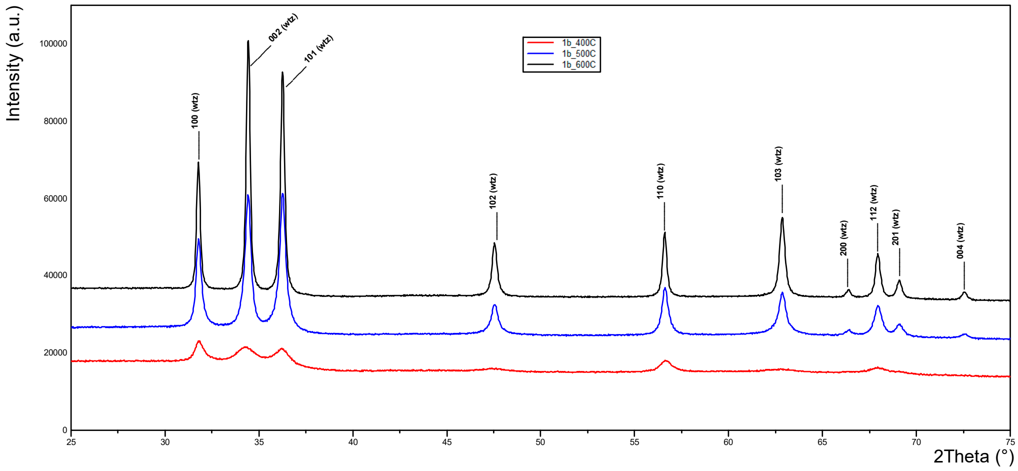

Figure S2: Indexed XRD patterns, in the main angular range from 25 to 75 deg 2θ, of the ZnO films **1b** obtained at 400, 500, and 600 °C.

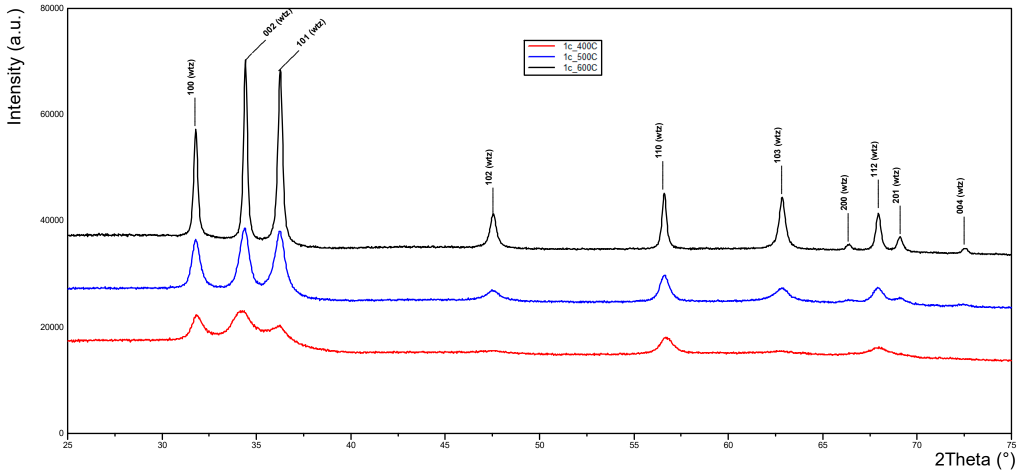

Figure S3: Indexed XRD patterns, in the main angular range from 25 to 75 deg 2θ, of the ZnO films **1c** obtained at 400, 500, and 600 °C.

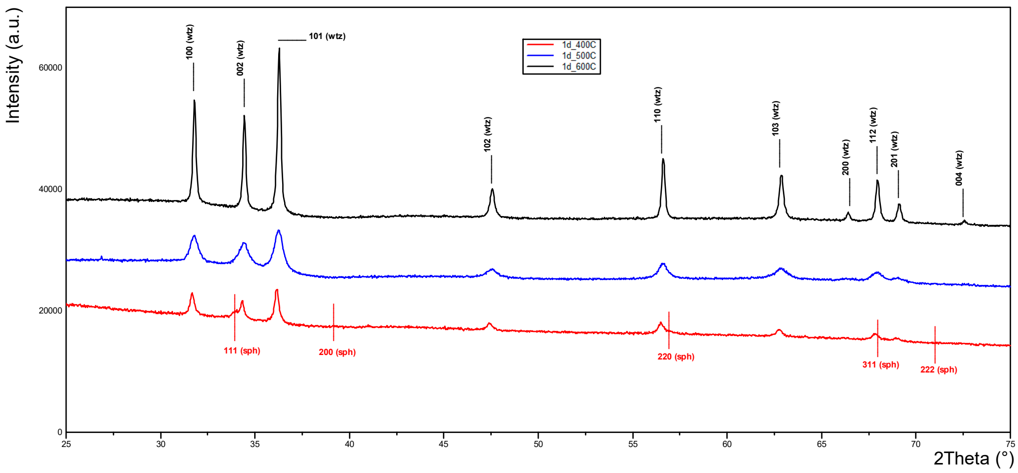

Figure S4: Indexed XRD patterns, in the main angular range from 25 to 75 deg 2θ, of the ZnO films **1d** obtained at 400, 500, and 600 °C.

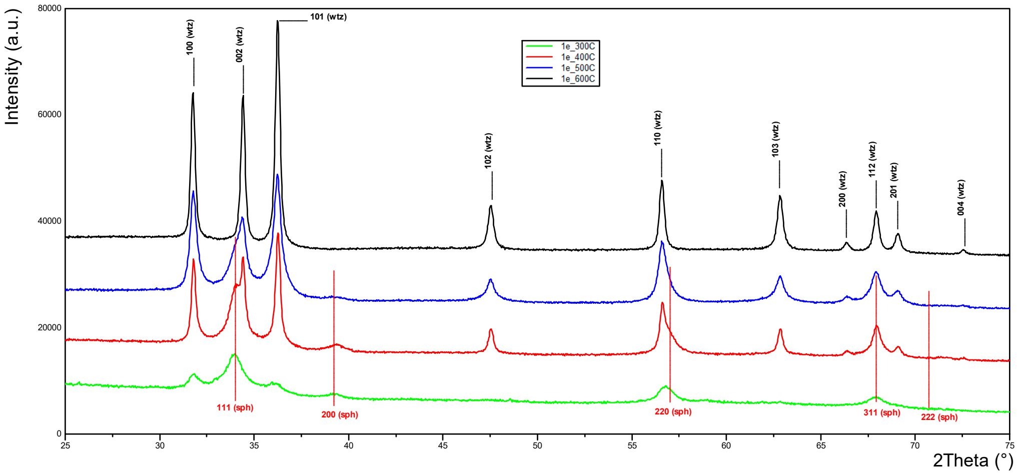

Figure S5: Indexed XRD patterns, in the main angular range from 25 to 75 deg 2θ, of the ZnO films **1e** obtained at 300, 400, 500, and 600 °C.

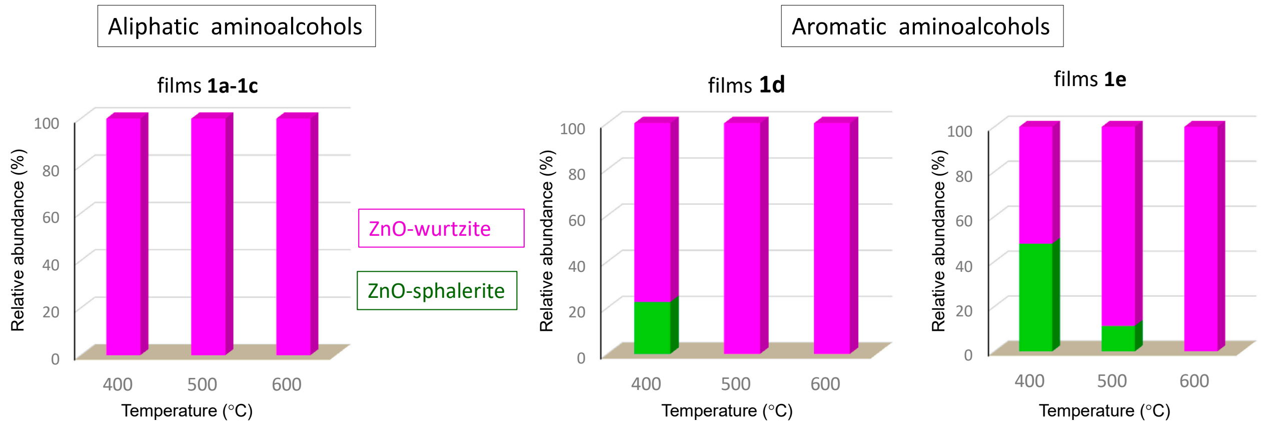

Figure S6: Variation of the relative abundance of the sphalerite and wurtzite forms of ZnO present in films **1a–1e** obtained after the thermal treatment at 400, 500 and 600 °C.

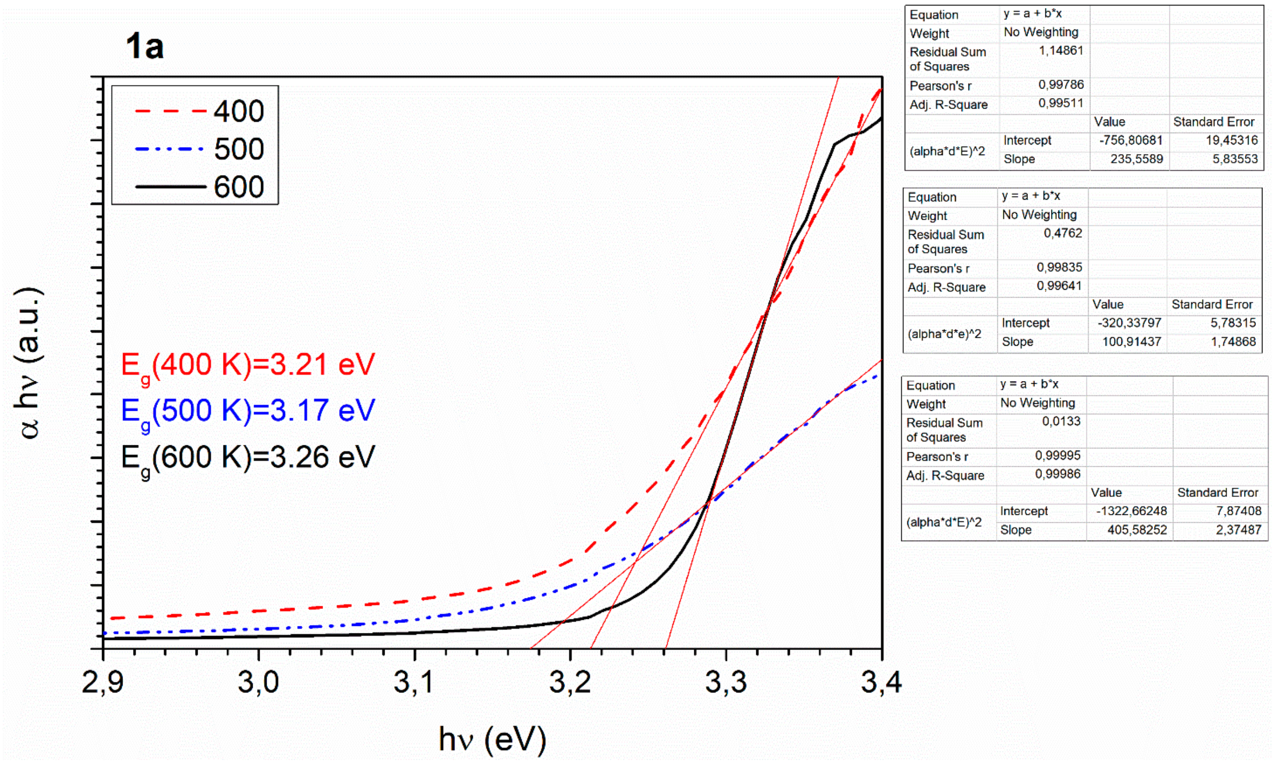

Figure S7: Tauc's plot for films **1a** at each annealing temperature.

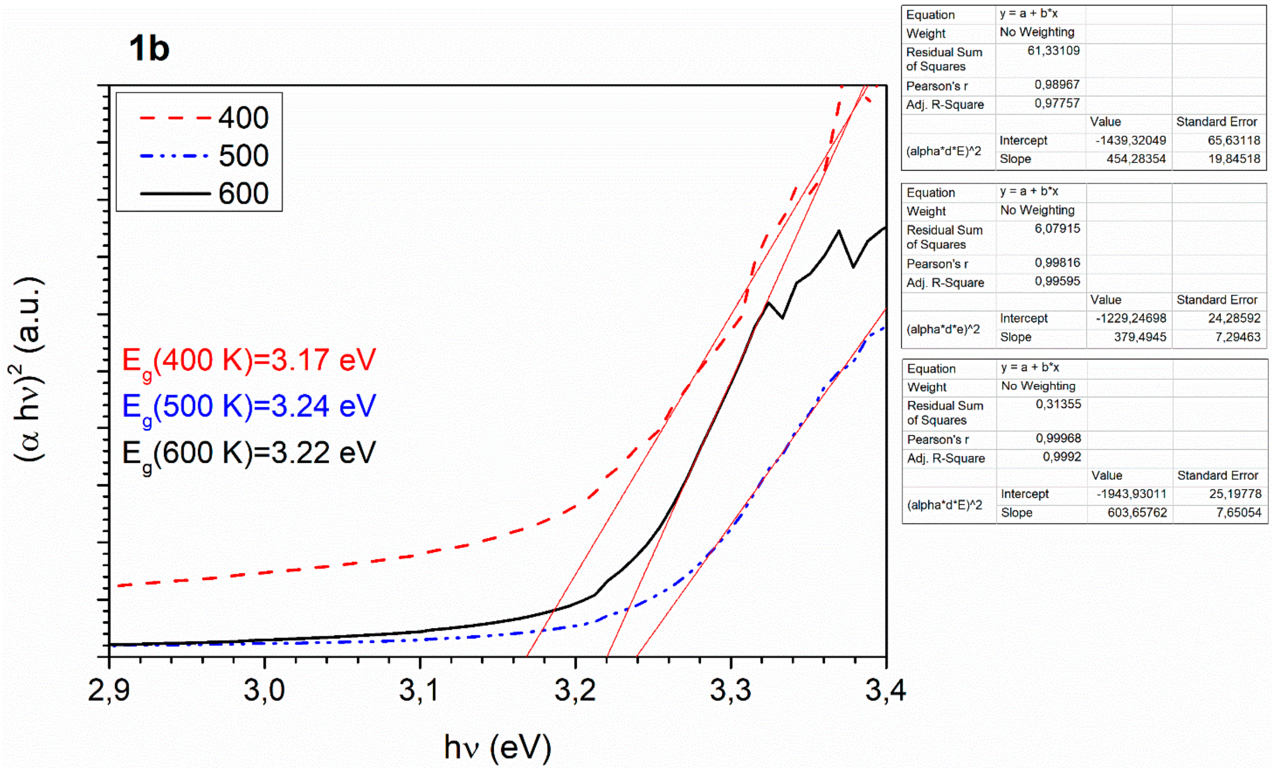

Figure S8: Tauc's plot for films **1b** at each annealing temperature.

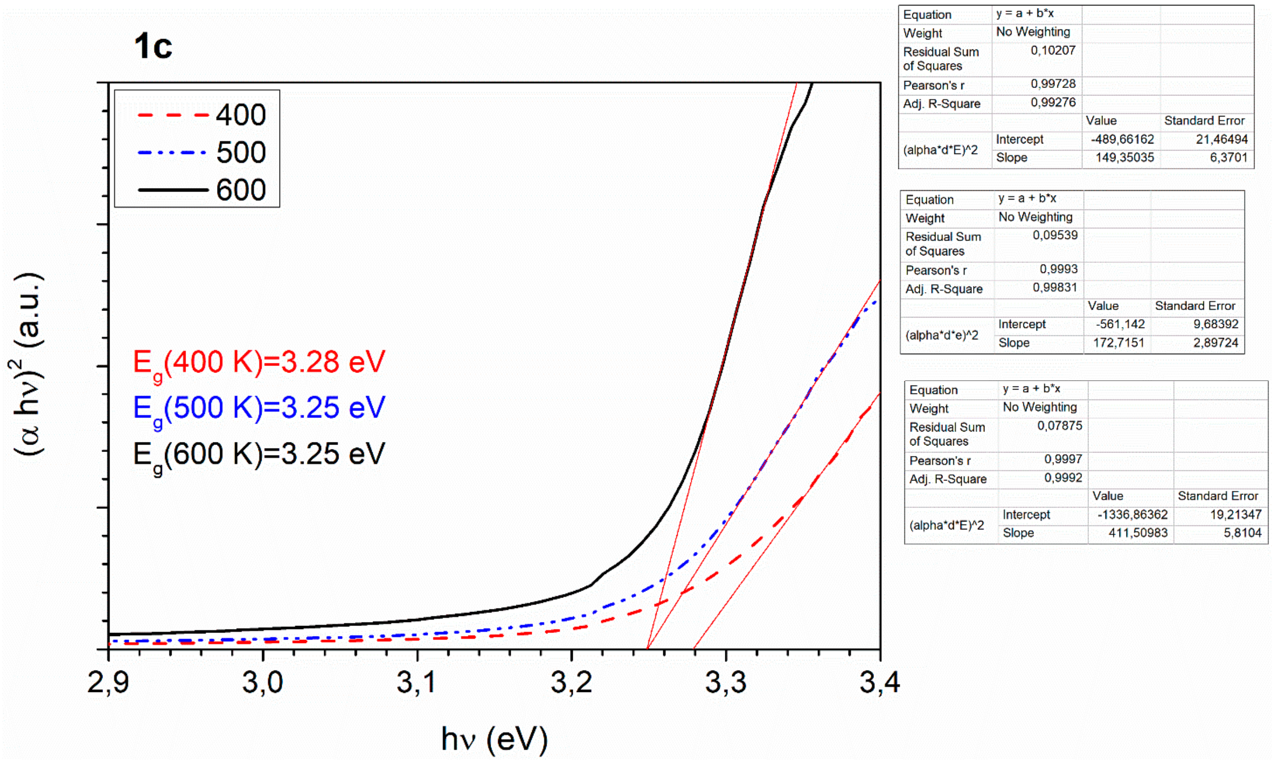

Figure S9: Tauc's plot for films **1c** at each annealing temperature.

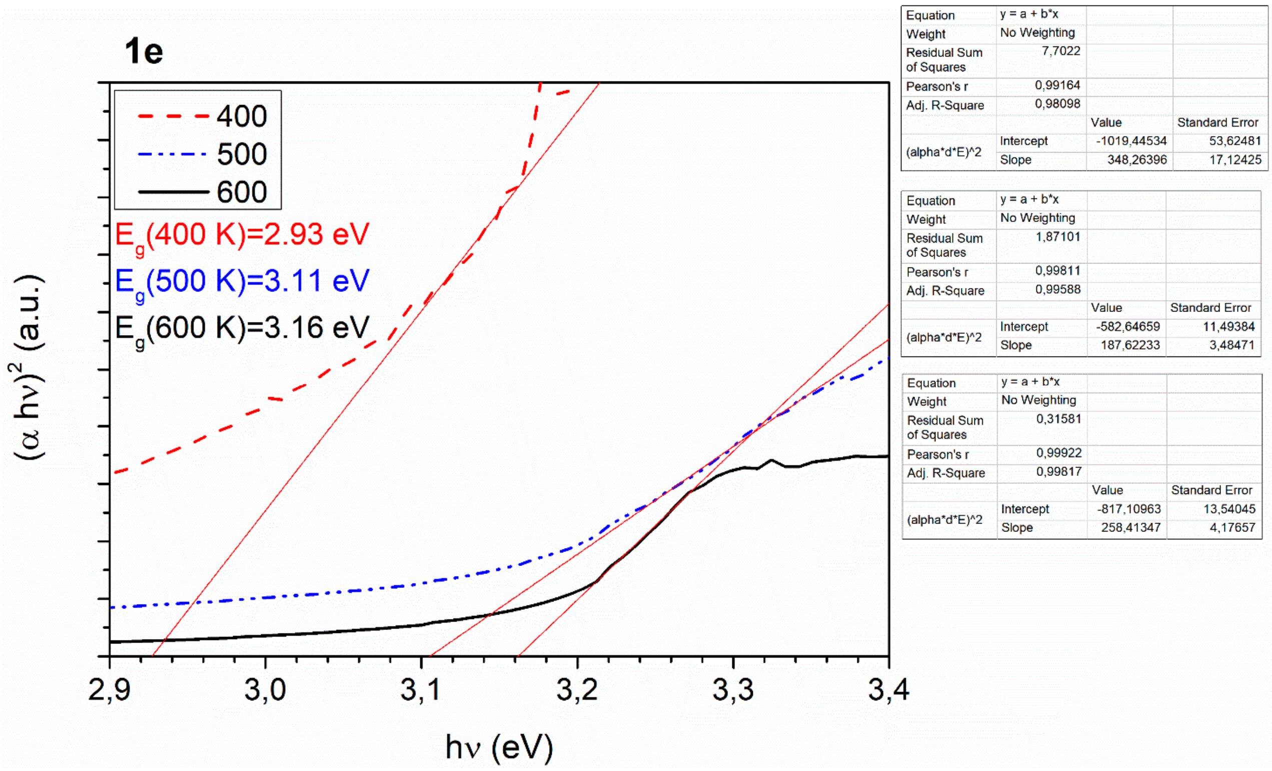

Figure S10: Tauc's plot for films **1e** at each annealing temperature.

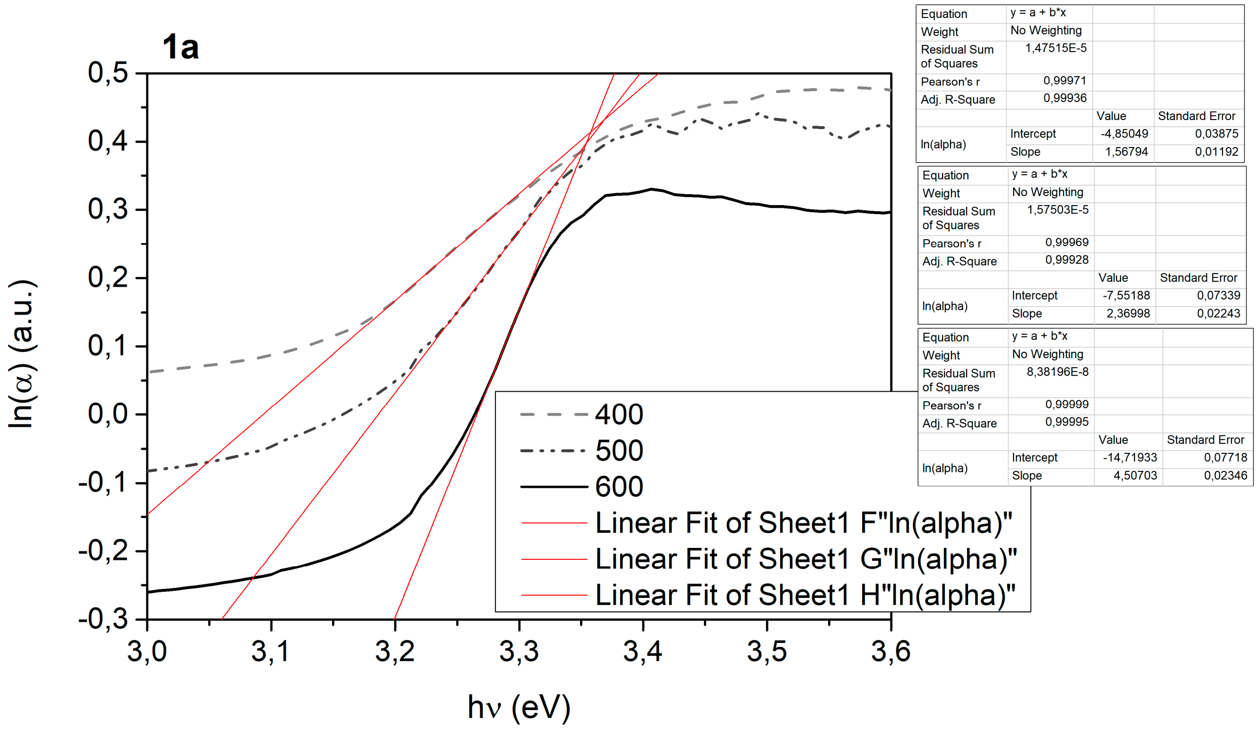

Figure S11: Fittings to find the Urbach's energy of films **1a** at each annealing temperature;

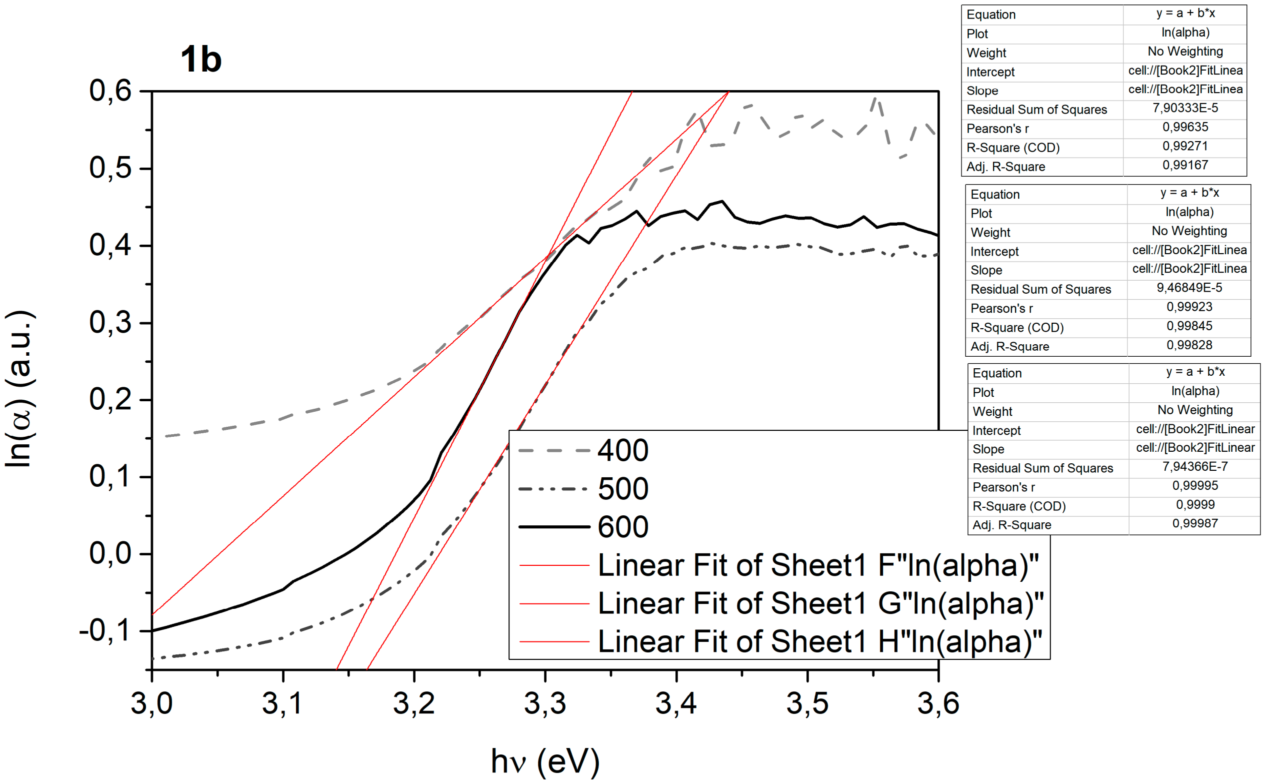

Figure S12: Fittings to find the Urbach's energy of films **1b** at each annealing temperature;

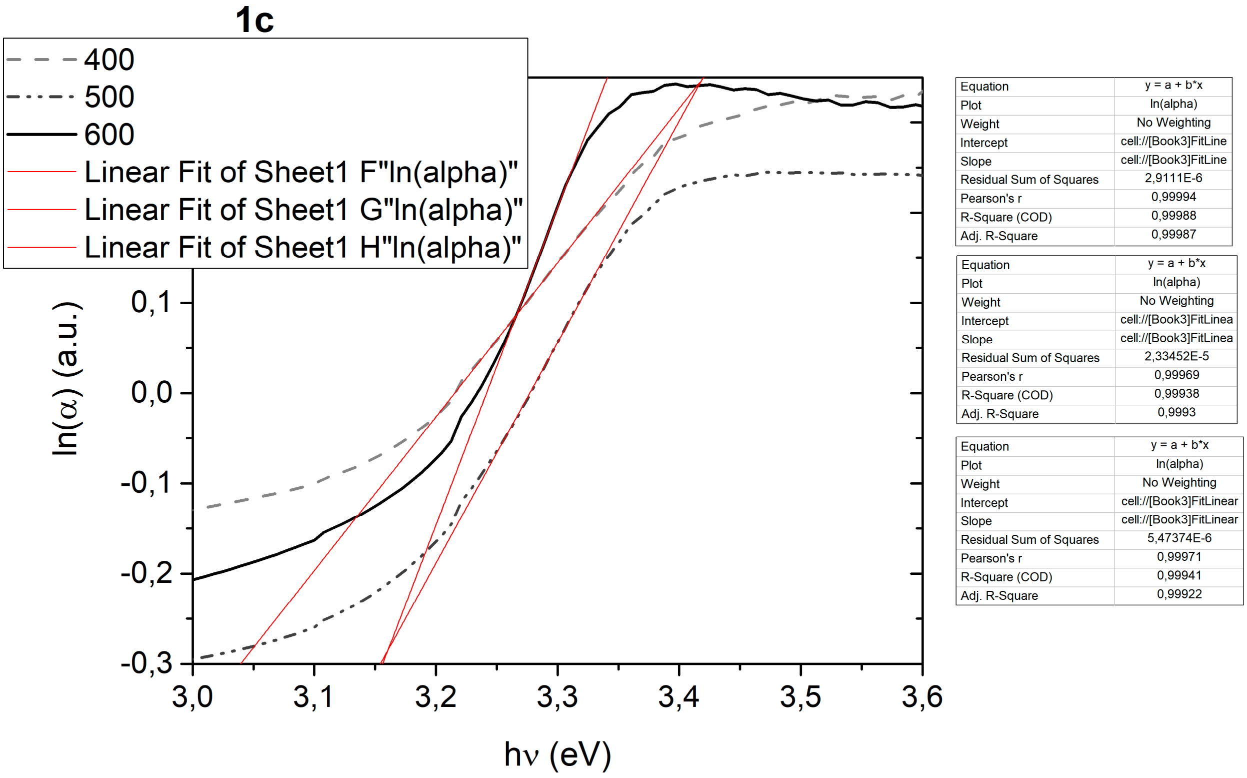

Figure S13: Fittings to find the Urbach's energy of films **1c** at each annealing temperature;

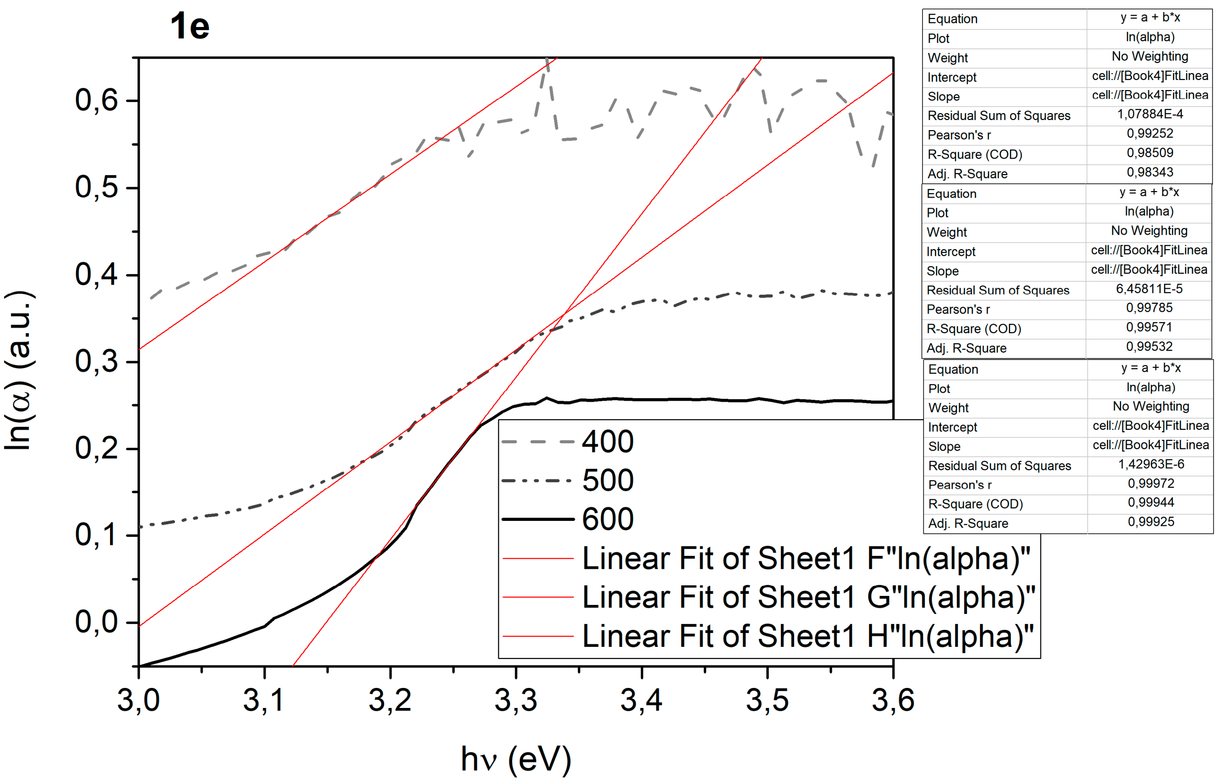

Equation	y = a + b*x
Plot	ln(alpha)
Weight	No Weighting
Intercept	cell://[Book4]FitLinea
Slope	cell://[Book4]FitLinea
Residual Sum of Squares	1,07884E-4
Pearson's r	0,99252
R-Square (COD)	0,98509
Adj. R-Square	0,98343
Equation	y = a + b*x
Plot	ln(alpha)
Weight	No Weighting
Intercept	cell://[Book4]FitLinea
Slope	cell://[Book4]FitLinea
Residual Sum of Squares	6,45811E-5
Pearson's r	0,99785
R-Square (COD)	0,99571
Adj. R-Square	0,99532
Equation	y = a + b*x
Plot	ln(alpha)
Weight	No Weighting
Intercept	cell://[Book4]FitLinea
Slope	cell://[Book4]FitLinea
Residual Sum of Squares	1,42963E-6
Pearson's r	0,99972
R-Square (COD)	0,99944
Adj. R-Square	0,99925

Figure S14: Fittings to find the Urbach's energy of films **1e** at each annealing temperature;

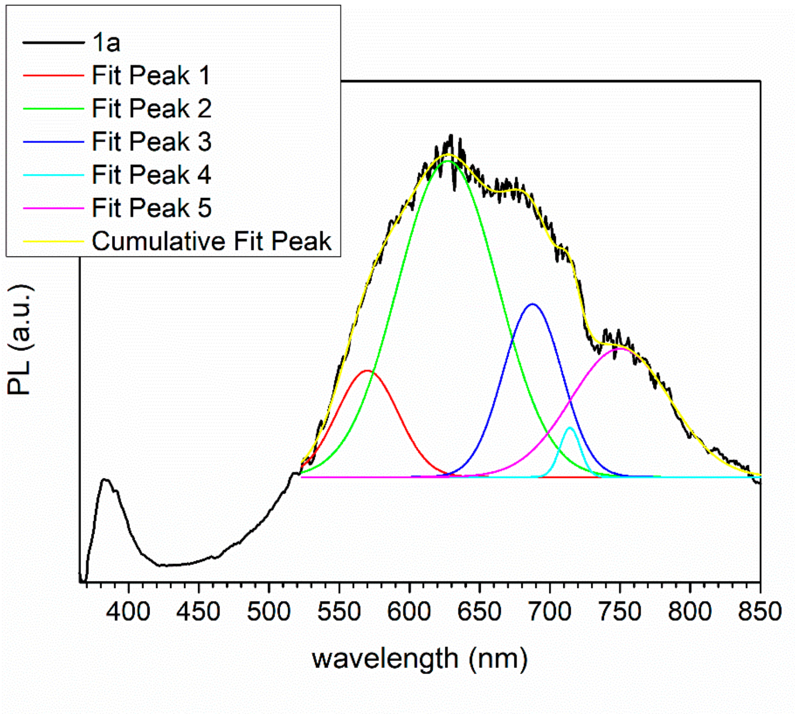

Model	Gauss		
Equation	y=y0 + (A/(w*sqrt(PI/2)))*exp(-2*((x-xc)/w)^2)		
Reduced Chi-Sqr	7,86281E11		
Adj. R-Square	0,99524		
		Value	Standard Error
Peak1(PL)	y0	1,26115E7	276759,07217
Peak1(PL)	xc	570,01493	4,12038
Peak1(PL)	w	43,48967	7,00388
Peak1(PL)	A	6,94441E8	5,36704E8
Peak1(PL)	sigma	21,74483	3,50194
Peak1(PL)	FWHM	51,20517	8,24644
Peak1(PL)	Height	1,27406E7	7,79493E6
Peak2(PL)	y0	1,26115E7	276759,07217
Peak2(PL)	xc	627,26229	1,74822
Peak2(PL)	w	71,52891	18,93273
Peak2(PL)	A	3,39227E9	1,02075E9
Peak2(PL)	sigma	35,76445	9,46636
Peak2(PL)	FWHM	84,21885	22,29158
Peak2(PL)	Height	3,78398E7	1,01458E6
Peak3(PL)	y0	1,26115E7	276759,07217
Peak3(PL)	xc	687,58711	1,97905
Peak3(PL)	w	42,10282	6,22919
Peak3(PL)	A	1,09215E9	5,38831E8
Peak3(PL)	sigma	21,05141	3,11459
Peak3(PL)	FWHM	49,57229	7,33431
Peak3(PL)	Height	2,06972E7	7,14101E6
Peak4(PL)	y0	1,26115E7	276759,07217
Peak4(PL)	xc	714,39476	0,54862
Peak4(PL)	w	14,53748	1,98594
Peak4(PL)	A	1,07415E8	3,22452E7
Peak4(PL)	sigma	7,26874	0,99297
Peak4(PL)	FWHM	17,11657	2,33827
Peak4(PL)	Height	5,89543E6	1,07681E6
Peak5(PL)	y0	1,26115E7	276759,07217
Peak5(PL)	xc	750,3206	2,19961
Peak5(PL)	w	70,69585	3,84977
Peak5(PL)	A	1,36096E9	1,07402E8
Peak5(PL)	sigma	35,34793	1,92488
Peak5(PL)	FWHM	83,23801	4,53276
Peak5(PL)	Height	1,53601E7	421928,63131

Figure S15: PL spectrum of film **1a** annealed at 600 °C and Gaussian deconvolution of its DLE bands.

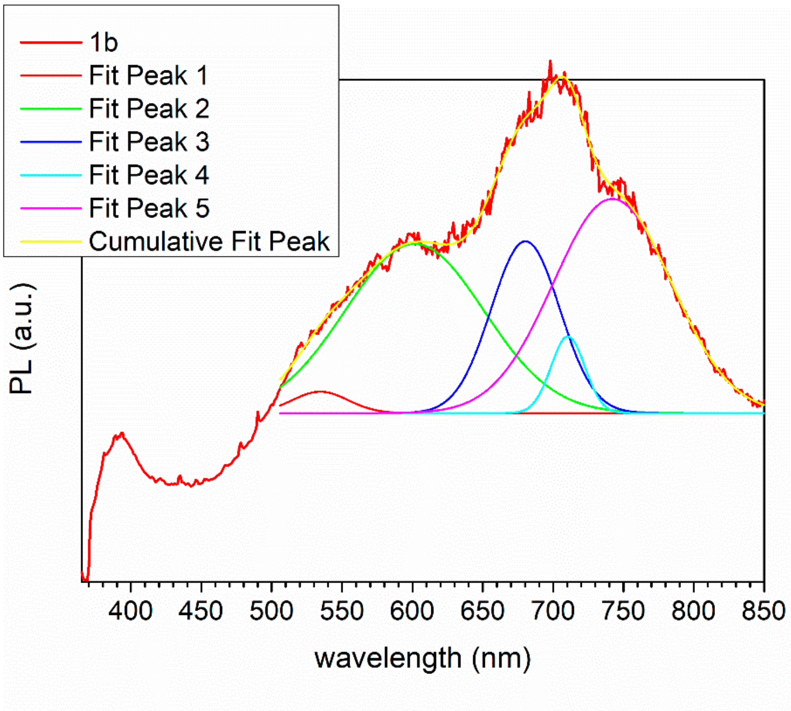

Model	Gauss		
Equation	y=y0 + (A/(w*sqrt(PI/2)))*exp(-2*((x-xc)/w)^2)		
Reduced Chi-Sqr	7,9052E11		
Adj. R-Square	0,99325		
		Value	Standard Error
Peak1(PL)	y0	2,0156E7	436323,01889
Peak1(PL)	xc	534,46719	3,4851
Peak1(PL)	w	39,57864	26,66308
Peak1(PL)	A	1,26812E8	2,69145E8
Peak1(PL)	sigma	19,78932	13,33154
Peak1(PL)	FWHM	46,60029	31,39338
Peak1(PL)	Height	2,55646E6	3,73673E6
Peak2(PL)	y0	2,0156E7	436323,01889
Peak2(PL)	xc	601,47592	2,32215
Peak2(PL)	w	98,45971	25,99971
Peak2(PL)	A	2,49748E9	6,88894E8
Peak2(PL)	sigma	49,22986	12,99986
Peak2(PL)	FWHM	115,92745	30,61232
Peak2(PL)	Height	2,02387E7	474160,08164
Peak3(PL)	y0	2,0156E7	436323,01889
Peak3(PL)	xc	680,31469	2,4642
Peak3(PL)	w	48,82599	7,60869
Peak3(PL)	A	1,25642E9	5,12387E8
Peak3(PL)	sigma	24,41299	3,80435
Peak3(PL)	FWHM	57,48821	8,95855
Peak3(PL)	Height	2,05317E7	5,45797E6
Peak4(PL)	y0	2,0156E7	436323,01889
Peak4(PL)	xc	710,51939	0,86429
Peak4(PL)	w	24,20228	2,85509
Peak4(PL)	A	2,7767E8	1,0847E8
Peak4(PL)	sigma	12,10114	1,42755
Peak4(PL)	FWHM	28,49601	3,36161
Peak4(PL)	Height	9,15405E6	2,56541E6
Peak5(PL)	y0	2,0156E7	436323,01889
Peak5(PL)	xc	742,13852	2,97694
Peak5(PL)	w	84,26422	4,59696
Peak5(PL)	A	2,70406E9	2,38985E8
Peak5(PL)	sigma	42,13211	2,29848
Peak5(PL)	FWHM	99,21353	5,41251
Peak5(PL)	Height	2,56043E7	1,00814E6

Figure S16: PL spectrum of film **1b** annealed at 600 °C and Gaussian deconvolution of its DLE bands.

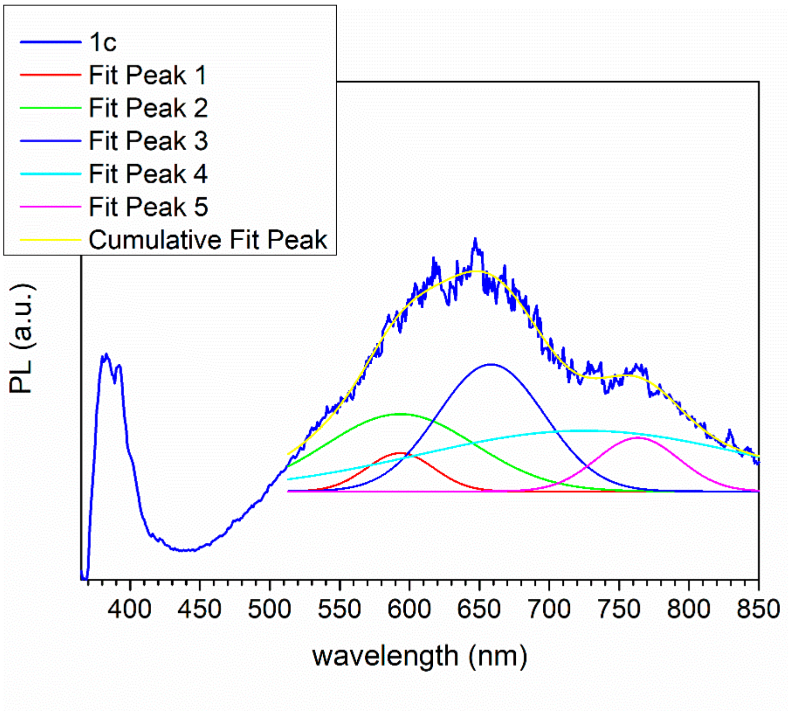

Model	Gauss		
Equation	y=y0 + (A/(w*sqrt(PI/2)))*exp(-2*((x-xc)/w)^2)		
Reduced Chi-Sqr	1,24026E12		
Adj. R-Square	0,97571		
		Value	Standard Error
Peak1(PL)	y0	1,06616E7	1,49295E8
Peak1(PL)	xc	593,31231	18,603
Peak1(PL)	w	48,23002	137,98241
Peak1(PL)	A	2,78533E8	5,89986E9
Peak1(PL)	sigma	24,11501	68,99121
Peak1(PL)	FWHM	56,7865	162,46187
Peak1(PL)	Height	4,60787E6	8,44873E7
Peak2(PL)	y0	1,06616E7	1,49295E8
Peak2(PL)	xc	593,31231	2709,82523
Peak2(PL)	w	106,77702	1709,53417
Peak2(PL)	A	1,24545E9	4,91921E10
Peak2(PL)	sigma	53,38851	854,76708
Peak2(PL)	FWHM	125,72034	2012,82266
Peak2(PL)	Height	9,30656E6	2,22428E8
Peak3(PL)	y0	1,06616E7	1,49295E8
Peak3(PL)	xc	658,22444	392,05166
Peak3(PL)	w	76,41731	543,94666
Peak3(PL)	A	1,46364E9	6,77722E10
Peak3(PL)	sigma	38,20865	271,97333
Peak3(PL)	FWHM	89,97451	640,44825
Peak3(PL)	Height	1,52821E7	5,94698E8
Peak4(PL)	y0	1,06616E7	1,49295E8
Peak4(PL)	xc	724,9452	2367,76184
Peak4(PL)	w	235,80196	6228,70941
Peak4(PL)	A	2,15782E9	1,15482E11
Peak4(PL)	sigma	117,90098	3114,35471
Peak4(PL)	FWHM	277,63559	7333,74489
Peak4(PL)	Height	7,30144E6	1,98112E8
Peak5(PL)	y0	1,06616E7	1,49295E8
Peak5(PL)	xc	763,36151	32,77046
Peak5(PL)	w	58,94721	28,39006
Peak5(PL)	A	4,75919E8	1,13677E9
Peak5(PL)	sigma	29,4736	14,19503
Peak5(PL)	FWHM	69,40504	33,42674
Peak5(PL)	Height	6,44184E6	1,24677E7

Figure S17: PL spectrum of film **1c** annealed at 600 °C and Gaussian deconvolution of its DLE bands.

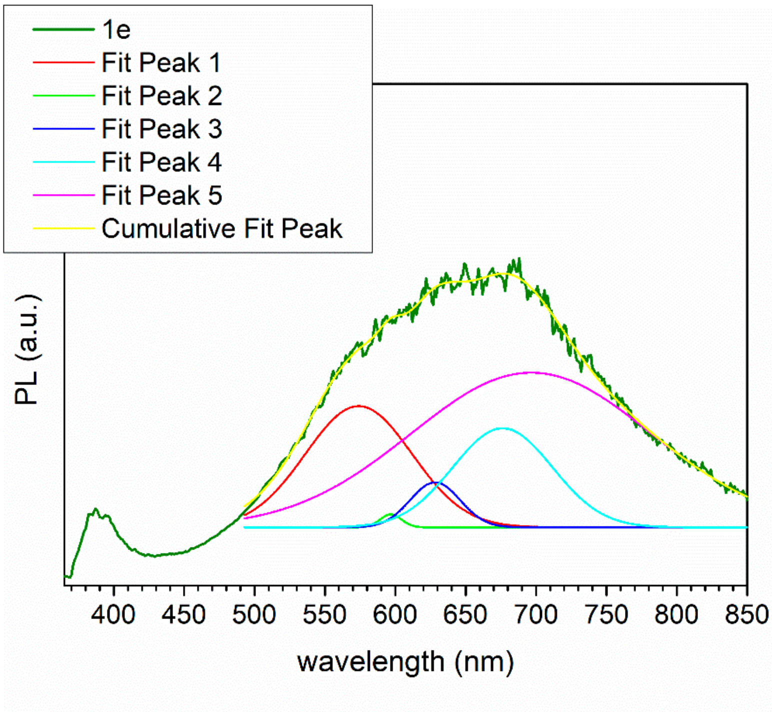

Model	Gauss		
Equation	y=y0 + (A/(w*sqrt(PI/2)))*exp(-2*((x-xc)/w)^2)		
Reduced Chi-Sqr	5,65824E11		
Adj. R-Square	0,99404		
		Value	Standard Error
Peak1(PL)	y0	6,40872E6	3,36137E6
Peak1(PL)	xc	574,04623	11,83957
Peak1(PL)	w	75,7204	25,63561
Peak1(PL)	A	1,46726E9	2,05457E9
Peak1(PL)	sigma	37,8602	12,81781
Peak1(PL)	FWHM	89,15396	30,18363
Peak1(PL)	Height	1,54609E7	1,67109E7
Peak2(PL)	y0	6,40872E6	3,36137E6
Peak2(PL)	xc	596,86595	2,05293
Peak2(PL)	w	15,23788	5,27641
Peak2(PL)	A	3,1629E7	2,1068E7
Peak2(PL)	sigma	7,61894	2,63821
Peak2(PL)	FWHM	17,94123	6,2125
Peak2(PL)	Height	1,65616E6	641181,71346
Peak3(PL)	y0	6,40872E6	3,36137E6
Peak3(PL)	xc	628,36757	1,9094
Peak3(PL)	w	35,47363	5,61735
Peak3(PL)	A	2,55264E8	1,1138E8
Peak3(PL)	sigma	17,73681	2,80868
Peak3(PL)	FWHM	41,767	6,61393
Peak3(PL)	Height	5,74148E6	1,72232E6
Peak4(PL)	y0	6,40872E6	3,36137E6
Peak4(PL)	xc	676,28732	9,30366
Peak4(PL)	w	70,66108	11,61413
Peak4(PL)	A	1,11896E9	1,1429E9
Peak4(PL)	sigma	35,33054	5,80707
Peak4(PL)	FWHM	83,19706	13,67459
Peak4(PL)	Height	1,2635E7	1,10126E7
Peak5(PL)	y0	6,40872E6	3,36137E6
Peak5(PL)	xc	696,6194	71,23541
Peak5(PL)	w	170,42307	88,79925
Peak5(PL)	A	4,21868E9	4,74706E9
Peak5(PL)	sigma	85,21154	44,39963
Peak5(PL)	FWHM	200,65783	104,55313
Peak5(PL)	Height	1,9751E7	1,14986E7

Figure S18: PL spectrum of film **1e** annealed at 600 °C and Gaussian deconvolution of its DLE bands.

Table S1: Wavelet position of each NBE component obtained after Gaussian decovolution for **1a–1c** and **1e** films annealed at 600 °C.

Aminoalcohol	Aliphatic			Aromatic
Film	**1a**	**1b**	**1c**	**1e**
Calculated maximum wavelength (nm)	381	373[a]	380	384
	391	392	391	395

[a] Rather insignificant in comparison to the other peak
